# Effect of Blueberry Extract on Liver in Aged Rats

**DOI:** 10.1155/2022/3490776

**Published:** 2022-07-18

**Authors:** Tarfa Albrahim, Mona Alonazi

**Affiliations:** ^1^Department of Health Sciences, Clinical Nutrition, College of Health and Rehabilitation Sciences, Princess Nourah Bint Abdulrahman University, P.O. Box 84428, Riyadh 11671, Saudi Arabia; ^2^Biochemistry Department, Science College, King Saud University, P.O. Box 22452, Riyadh 11495, Saudi Arabia

## Abstract

Aging and age-related disorders are prominent issues. Aging is associated with a gradual impairment of physiology at the genetic, cellular, tissue, and whole organism level that directly influences the development of chronic diseases and organ failure. Blueberries, on the other hand, are well known for their high content of bioactive compounds and have demonstrated positive impacts on metabolic factors that influence health and general well-being. This study is aimed at evaluating the ameliorating the effects of blueberry on the liver of aged rats by monitoring changes in metabolic disturbances, oxidative stress, and inflammatory disruption. The aged group of rats was orally administered with blueberry extract (200 mg/kg) for a period of 4 weeks. The results revealed that aging was associated with an increase in body weight, liver weight, and metabolic parameters like serum insulin, triglycerides, total cholesterol, and liver function markers accompanied with a decrease in vitamin D levels. Furthermore, the results showed a significant diminish in the activities of antioxidant enzymes, glutathione content with an elevation in lipid peroxidation, inflammatory mediators (tumor necrosis factor alpha, interleukin 6, and nuclear factor kappa-light-chain-enhancer of activated B cells) as well as fibrotic markers (TGF-*β*1) in the liver of aged rats. Compared to the young rats (control group), blueberry effectively reversed age-mediated disruption of the aforementioned parameters. Hence, blueberries can be used as a potential therapeutic strategy for the management of age-related liver dysfunction and disease.

## 1. Introduction

Aging and age-related disorders have become one of the foremost issues globally. Aging is accompanied by a gradual impairment of physiology at genetic, cellular, tissue, and entire organism levels [1]. Aging directly influences the development of chronic diseases and organ failure. The liver is a fundamental organ for lipogenesis, gluconeogenesis, and cholesterol metabolism. Although the liver exhibits a marked resilience, the aging process is strongly linked to a number of degenerative changes in the liver, including a reduction in hepatic structure and cell function [1]. Many studies suggest that oxidative stress and inflammatory dysregulation play a key role in tissue damage and function associated with aging, among other known factors, although the precise mechanism remains unclear [2, 3].

Free radical hypothesis is one of the most frequently accepted explanations for senescence among aging theories. Hence, a healthy lifestyle including proper nutrition as well as physical activity is well known to slow down the process of aging. In the past years, there has been considerable interest in the use of natural plant-based diets and their positive impacts on the aging process. Fruit and vegetable consumption has an inverse association with the occurrence of cardiovascular illnesses, cancer, and other chronic diseases, according to clinical trials and epidemiological studies [4]. Blueberries, because of their enriched nutritive contents, have particularly attracted a lot of attention in this regard [5].

Blueberry is a member of the Ericaceae family's Vaccinium genus, which has about 450 species worldwide [6]. Blueberry fruits are low in calories and high in water content, micronutrients, prebiotic fibers, vitamins, sugar units, and other acidic moieties which are often loaded with many bioactive polyphenols (PP) [7, 8]. PP is the secondary metabolite of plants and constitutes a diverse group of compounds classified as flavonoids, phenolic acids, chalcones, and coumarins as well as polymers [9, 10]. The physicochemical properties of PP, their bioaccessibility, cellular uptake, metabolism, and transport determine their availability and consequently their biological effects [11]. Interestingly, anthocyanins were found in the liver, eye, brain, and cerebellum of pigs fed wild blueberries for four weeks [12], whereas flavanol levels were higher in both plasma and brain tissue of aged rats treated with blueberries for 12 weeks, according to Williams et al. [13].

Blueberries are well known for their high antioxidant capacity with a high concentration of anthocyanins as well as other phenolic compounds and have been observed to reduce the risk of diabetes [14], obesity [15], aging [16], urinary tract infections [17], cancer [18, 19], etc. In this regard, Papandreou et al. [16] reported that blueberry supplementation improved cognition and inhibited acetylcholinesterase activity in aged rats. However, no previous study was employed to evaluate the effect of blueberry in the liver of aged rats. Thus, this study was aimed at searching for the beneficial effects of consuming blueberry extract on metabolic factors that influence health with particular emphasis on the liver of aged rats.

## 2. Materials and Methods

### 2.1. Blueberry Extraction

A local market in Riyadh, Saudi Arabia, provided fresh blueberry fruits. The blueberry fruits were confirmed by a professional taxonomist from Saudi Arabia's Princess Nourah Bint Abdulrahman University. The fruits were washed, then mashed into juice, and macerated in methanol (80%; *v*/*v*) for 24 hours at 4°C. The macerated product was filtered, and the resultant fluid was condensed to a semidry state before being dissolved in distilled water using a rotary evaporator. The extract collected was labelled as blueberry extract (BBE). Total phenolics, flavonoids, and anthocyanins were measured using standard methods, with the results reported as gallic acid equivalents (GAE), quercetin equivalents (QE), and cyanidin 3-rutinoside equivalents per gram of dry extract, respectively.

### 2.2. Animals

Six-month-old (control group) and twenty-four-month-old (group aged and group aged treated with blueberry extract; aged+BBE) male Wistar rats were used in the present study. The rats were fed standard rat chow (2016 Teklad global 16 percent protein rodent diets; Envigo, USA), had free access to water, and were treated humanely in accordance with the animal care provisions. They were housed in temperature- and humidity-controlled animal quarters with a 12-hour light-dark cycle. Every day, the rats were weighed. The group aged+BBE got BBE 200 mg/kg bodyweight orally via gavage, whereas the control and aged groups received only saline. BBE dose (200 mg/kg) was chosen based on Debom et al.'s study [20]. The study ran for four weeks.

All experimental procedures were performed in accordance with Princess Nourah Bint Abdulrahman University Institutional Animal Care and Use Committee (IACUC) guidelines (approval no. HAP-01-R-059; IRB registration no.: 22-0161; category of approval: exempt).

### 2.3. Sampling and Tissue Preparation

After a one-month intervention period, rats were euthanized by an overdose of pentobarbital (300 mg/kg i.p.). A syringe puncture was used to take blood from the heart, which was then held at 37°C for 30 minutes before being centrifuged at 3000 x g for 10 minutes to extract the serum, which was then stored at 80°C for biochemical analysis. The liver was dissected and divided into three sections right away. The first piece of liver tissue was homogenized in a 10x volume of ice-cold 0.05 M potassium phosphate buffer (pH 7.4). The supernatant was separated by centrifugation at 3000 x g (4°C) for 10 minutes. Biochemical examination was performed on the supernatants, which were maintained at a temperature of 80 degrees Celsius. The remaining two liver parts were sampled and fixed in 10% buffered formalin for histopathological examination.

### 2.4. Serum Biochemical Parameters

A colorimetric assay kit (Biodiagnostics, Cairo, Egypt) was used to estimate serum glucose levels, while ELISA kits from Thermo Fisher Scientific (Waltham, MA, USA) were used to measure serum insulin levels, and 25-hydroxyvitamin D [25(OH)D] was measured using an ELISA kit from Abcam (Cambridge, UK) according to the manufacturer's instructions. RANDOX Reagents provided commercial kits to assess total cholesterol and triglycerides (USA). Alanine transaminase (ALT) and aspartate transaminase (AST) were also tested to evaluate liver function.

### 2.5. Oxidative Stress Markers

To assess biomarkers for oxidative stress, malondialdehyde (MDA) level was estimated based on the protocol of Ohkawa et al. [21]. In addition, reduced glutathione (GSH) contents was measured following the method of Ellman [22].

### 2.6. Antioxidant Enzymatic Activities

The methods described by Nishikimi et al. [23] and Aebi [24] were utilized for estimation of superoxide dismutase (SOD) and catalase (CAT) activities, correspondingly. Furthermore, the activities of glutathione peroxidase (GPx) were assayed according to Paglia and Valentine [25] and glutathione reductase (GR) was estimated based on the methods of De Vega et al. [26].

### 2.7. Determination of Inflammatory Markers

The levels of interleukin-6 (IL-6), tumor necrosis factor-*α* (TNF-*α*), and nuclear factor kappa-light-chain-enhancer of activated B cells p65 (NF-*κ*B p65) in liver tissue were measured by ELISA kits obtained from R&D Systems (Minneapolis, MN, USA) according to the manufacturer's instructions.

### 2.8. Determination of Hepatic Fibrosis Markers

The levels of transforming growth factor (TGF-*β*1) were measured using ELISA kit obtained from Abcam (Cambridge, UK) according to the manufacturer's protocol.

### 2.9. Histological Examination

Histological exams were used to evaluate liver tissue. In brief, liver tissues were fixed in buffered 10% formaldehyde before being implanted in paraffin. To assess general histological features, the embedded tissue samples were sectioned (5 *μ*m) and stained with hematoxylin and eosin.

### 2.10. Statistical Analysis

SPSS software was used to statistically evaluate all data. To determine whether there was a significant difference between groups, a one-way ANOVA was employed, followed by a Tukey's post hoc test. The results were presented as the mean ± standard deviation (SD), with a *p* value of less than 0.05 considered significant.

## 3. Results

### 3.1. Phytochemical Analysis of Blueberry Extract (BBE)

The results showed that BBE has a total polyphenolic content of 7.4 ± 0.1 mg GAE/g dry weight, flavonoids content of 4.1 ± 0.08 mg QE/g dry weight, and total anthocyanin content of 1.7 ± 0.06 mg cyanidin 3-rutinoside equivalents/g dry weight.

### 3.2. Effect of Blueberry Extract (BBE) on Body Weight, Liver Weight, and Liver Ratio

**Figure 1:**
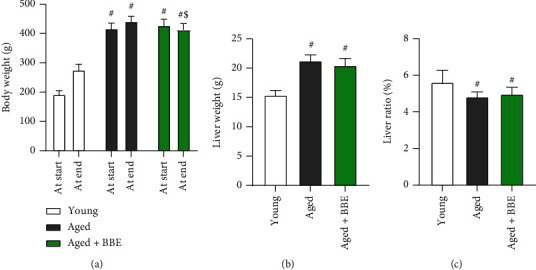
Effect of blue berry extract on (a) body weight change, (b) liver weight, and liver ratio (c) in aged rats. Data are expressed as the mean ± SD (*n* = 7). ^#^ and ^$^ indicate statistically significant differences between young rats and aged rats, respectively, at *p* < 0.05.

Following the intervention period of 4 weeks, aged rats showed an increase in their body weight and liver weight, while the aged+BBE group treated with blueberry extract at 200 mg/kg showed a decline in both body weight and liver weight (*p* < 0.05) compared to the aged rats (Figure 1). In contrast, the liver ratio notably decreased in the aged and aged+BBE groups when compared to the young rats; however, no significant difference in the liver ratio was observed in the group treated with blueberry extract (aged+BBE rats) and untreated aged rats.

### 3.3. Effect of Blue**berry** Extract on Liver Function Parameters

**Figure 2:**
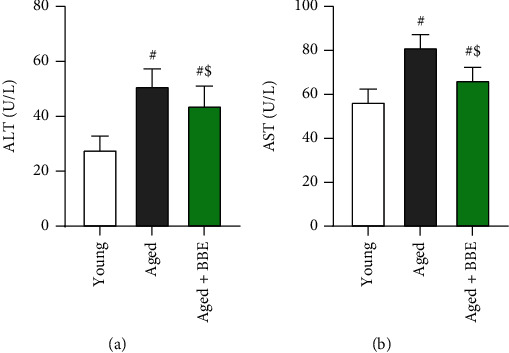
Effect of blue berry extract on liver function parameters: (a) ALT and (b) AST in aged rats. Data are expressed as the mean ± SD (*n* = 7). ^#^ and ^$^ indicate statistically significant differences between young rats and aged rats, respectively, at *p* < 0.05.

The levels of ALT and AST were measured for assessment of liver function. The aged rat group displayed marked elevations (*p* < 0.05) of both ALT and AST markers (Figure 2). However, administration with blueberry extract significantly reduced (*p* < 0.05) both hepatic function markers relative to the aged group.

### 3.4. Effect of Blueberry Extract on Serum Glucose, Insulin, Vitamin D, and Lipid Profile

**Figure 3:**
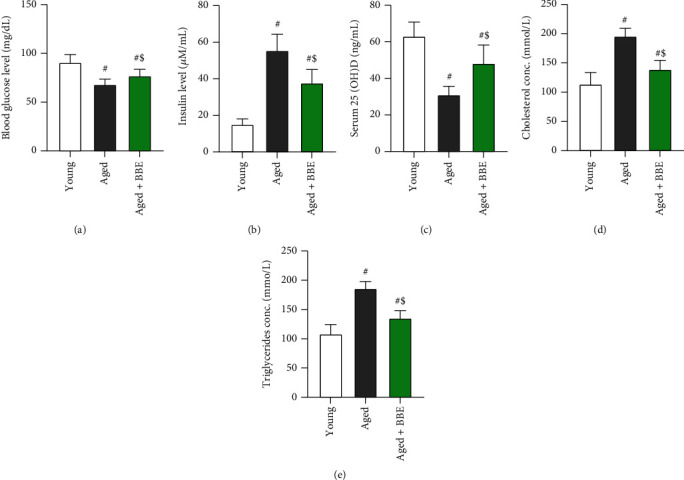
Effect of blue berry extract on serum levels of (a) glucose, (b) insulin, (c) 25(OH)D, (d) cholesterol, and (e) triglyceride in aged rats. Data are expressed as the mean ± SD (*n* = 7). ^#^ and ^$^ indicate statistically significant differences between young rats and aged rats, respectively, at *p* < 0.05.

Marked rises (*p* < 0.05) were noticed in serum levels of insulin, total cholesterol, and triglycerides, accompanied by a decline in blood glucose and 25(OH)D levels in the aged group compared to the young rats (Figure 3). However, supplementation of blueberry extract in the aged+BBE group significantly lowered the levels of serum insulin, total cholesterol, and triglycerides while notably increasing the levels of serum 25(OH)D and blood glucose (*p* < 0.05) when compared to the aged group.

### 3.5. Effect of Blueberry Extract on Hepatic Oxidative/Antioxidant Status

**Figure 4:**
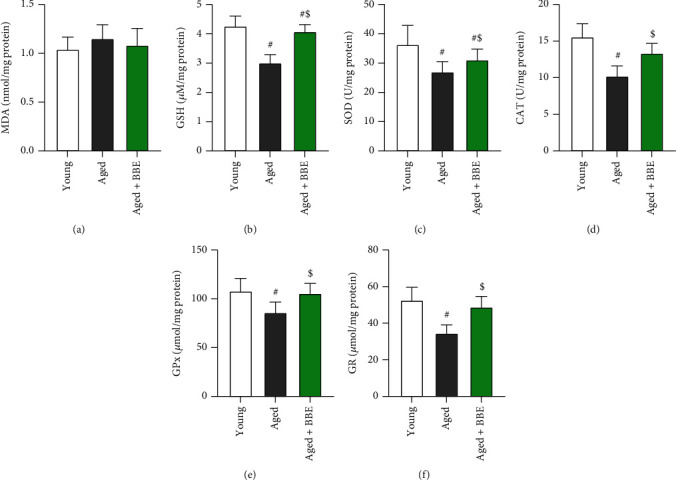
Effect of blue berry extract on hepatic levels of (a) malondialdehyde (a key marker for lipid peroxidation) and (b) glutathione and activities of (c) superoxide dismutase, (d) catalase, (e) glutathione peroxidase, and (f) glutathione reductase in aged rats. Data are expressed as the mean ± SD (*n* = 7). ^#^ and ^$^ indicate statistically significant differences between young rats and aged rats, respectively, at *p* < 0.05.

As observed in Figure 4, hepatic levels of malondialdehyde (a biomarker for lipid peroxidation) elevated nonsignificantly in the aged group of rats, while the levels of reduced glutathione (GSH) as well as enzymatic activity of the antioxidant enzymes SOD, CAT, GPx, and GR significantly decline (*p* < 0.05) in comparison to the young group. However, treatment with BBE showed a prompt nonsignificant decrease in the levels of MDA, while a marked enhancement in the levels of GSH were observed. Moreover, a significant increase is witnessed in the enzymatic activities of SOD, CAT, GPx, and GR (*p* < 0.05) compared to the aged rats.

### 3.6. Effect of Blueberry Extract on Hepatic Inflammatory Biomarkers

**Figure 5:**
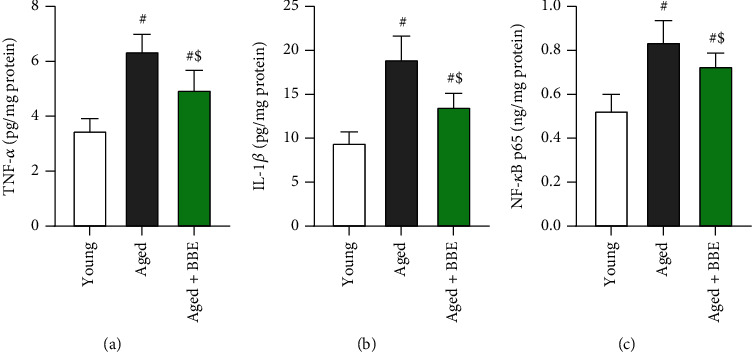
Effect of blue berry extract on hepatic levels of inflammatory markers: (a) tumor necrosis factor-*α*, (b) interleukin-1*β*, and (c) nuclear factor kappa-light-chain-enhancer of activated B cells in aged rats. Data are expressed as the mean ± SD (*n* = 7). ^#^ and ^$^ indicate statistically significant differences between young rats and aged rats, respectively, at *p* < 0.05.

The hepatic levels of inflammatory markers (tumor necrosis factor-*α*, interleukin-6, and nuclear factor kappa-light-chain-enhancer of activated B cells) (Figure 5) showed a marked increase with age (aged group); however, supplementation with blueberry extract remarkably ameliorated the levels of the aforementioned markers in hepatic tissues (*p* < 0.05) compared to the aged rats.

### 3.7. Effect of Blueberry Extract on Hepatic Fibrosis Markers

**Figure 6:**
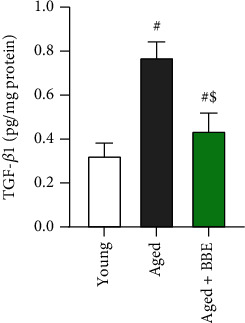
Effect of blue berry extract on hepatic transforming growth factor-beta in aged rats. Data are expressed as the mean ± SD (*n* = 7). ^#^ and ^$^ indicate statistically significant differences between young rats and aged rats, respectively, at *p* < 0.05.

Hepatic fibrosis was evaluated based on the levels of transforming growth factor (TGF-*β*1) (Figure 6). The markers showed a notable increase in the aged group of rats, which was significantly restored with administration of blueberry extract in the aged+BBE group (*p* < 0.05).

### 3.8. Histopathological Findings in the Liver

**Figure 7:**
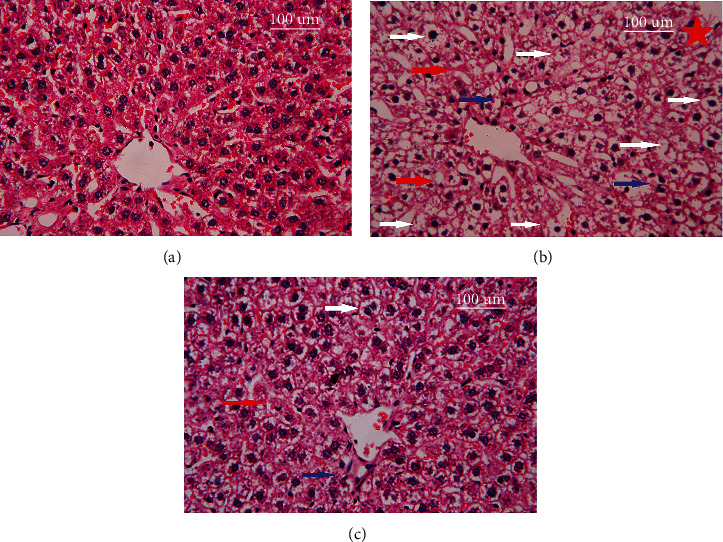
Photomicrographs of histological changes in the liver tissue following treatment with blue berry extract in aged rats. Scale bar 100 *μ*m. (a) Control young, (b) control aged, and (c) aged rats treated with blue berry extract. Red arrow: hepatocyte strand degeneration; blue arrow: diffused infiltration of inflammatory cells; and white arrow: vacuolated hepatocytes, and degeneration with focal area necrosis.

The liver sections from young (control) rats revealed a normal hepatocyte structure, where the hepatocytes are polygonal in shape with eosinophilic granular cytoplasm and vesicular basophilic nuclei (Figure 7(a)). A variety of major histopathological lesions were observed in the liver sections of aged rats showing marked derangement of hepatocyte strands, diffused infiltration of inflammatory cells, vacuolated hepatocytes, and degeneration with focal area necrosis (Figure 7(b)). On the other hand, liver sections of rats treated with blueberry extract revealed a moderate degree of improvement in hepatocytes, where there were only a few atrophied and/or vacuolated hepatocytes and a mild infiltration of inflammatory cells (Figure 7(c)).

## 4. Discussion

The effect of blueberry extract on the liver of aged rats was investigated in this study. The liver is the body's principal detoxifying organ, and it plays a critical role in maintaining energy and metabolic balance by regulating glucose and lipid metabolism processes [27, 28]. Aging leads to the progressive impairment of homeostasis at cellular, tissue, genomic, and whole-organism levels, thereby increasing the risk of disease and survival [1]. Age-related changes in liver function contribute to systemic susceptibility to age-related diseases. Blueberries are a rich source of phytochemicals, especially abundant in anthocyanins. In this regard, Loncaric et al. [29] reported that BBE contains catechin, epicatechin, procyanidin B1, caffeic acid, chlorogenic acid, 4-hydroxycinnamic acid, myricetin, quercetin, kaempferol, delphinidin, petunidin, cyanidin, peonidin, and malvidin. Anti-inflammatory and antioxidant properties, as well as favorable effects on glucoregulatory and vascular function, are among the most prominent health benefits of blueberries and/or anthocyanins, which have consequences in degenerative diseases and disorders, as well as the aging process.

The obtained results revealed a significant increase in both body weight as well as liver weight when compared to control rats; however, administration of BBE successfully reduced both body and liver weights in the aged+BBE group. In addition, supplementation with BBE modulated the high levels of serum triglycerides as well as total cholesterol, which is also in agreement with previous studies that suggest blueberries may positively affect serum biomarkers for lipid metabolism [30, 31]. Thus, the decrease in the size of animals after treatment may be due to ameliorating effects of blueberry on abnormal lipid metabolism. On the other hand, the liver ratio significantly diminished compared to the control group, and we noticed no significant difference between BBE administration and the aged group. This decline in the liver ratio may be due to the overall greater increase in body weight with aging when compared to liver weight. Liver function was also assessed based on the levels of AST and ALT in serum. Both AST and ALT function as endoenzymes in hepatocytes for amino acid synthesis as well as catabolism and are two of the most sensitive markers for liver cell damage [32, 33]. Results indicated elevation of both markers with aging; however, treatment with blueberry significantly reduced serum levels of both transaminases, suggesting a protective role of BBE against liver damage.

Our results showed a remarkable decline in serum insulin levels in rats receiving blueberry compared to the aged rats that did not receive treatment. On the other hand, fasting glucose levels were significantly reduced in the aged rats, and administration with blueberry successfully prevented it from reducing. This decline in fasting glucose may be due the effect of aging on the efficiency of intestinal absorption and/or food intake, but further studies should be considered to confirm our observation. However, several studies have shown positive effects of blueberry administration on both fasting glucose as well as serum insulin. *In vitro* studies with BBE in a cell culture-based bioassay, suggested its potential capacity to restrain B cell damage and improve insulin sensitivity [14]. Similarly, whole BBE afforded pancreatic B cell protection and prevented B cell apoptosis in another study on obese mice [34]. Likewise, a similar effect was observed for a blueberry leaf extract in pancreatic MIN6 B cells with improvement in insulin signaling while *in vivo* extract was able to decrease body weight, plasma glucose, hemoglobin A1c (HbA1c), homeostatic model assessment for insulin resistance (HOMA-IR), triglycerides, and nonesterified fatty acids (NEFAs) levels in mice fed with high-fat diet [35]. Moreover, in vivo clinical studies suggested improvements in type 2 diabetes mellitus and cardiometabolic parameters upon BBE consumption [30]. These studies are in agreement with our work and suggest blueberry and its bioactive compounds show their ameliorating effects by inducing increased expression of pancreatic *β* cell proliferation-related genes (*Ngn3*, *MafA*, *Pax4*, *Ins1*, and *Ins2*) and insulin signaling genes (IRS-1, IRS-2, PIK3ca, PDK1, PKC^″^, and GLUT-2), while downregulating FoxO1, a *β* cell apoptosis-related gene [35]. We also observed a decline in levels of 25(OH)D levels with advancing age. Vitamin D is a micronutrient that is metabolized into a secosteroid hormone essential for human health. Both 25 dihydroxy vitamin D [25(OH)2D] and its active hormonal form, 1,25-dihydroxy vitamin D [1,25(OH)2D] are necessary for human physiological functions and are key controllers of systemic inflammation, oxidative stress, and mitochondrial respiratory function, and thus, the aging process [36]. There has not been much study on the effects of blueberry on serum levels of vitamin D, and our results provide a novel therapeutic effect of blueberry whereby supplementation of BBE strongly improved serum levels of 25(OH)D. This observation suggests that blueberries may exert their positive effects by an overall improvement in metabolism.

To understand the effects of blueberry and its bioactive compounds on the process of aging, this study particularly focused on hepatic injury as a consequence of oxidative stress and inflammation using animal models. We measured endogenous MDA levels as well enzymatic antioxidants in the liver homogenates of aged rats to investigate the effects of aging linked to oxidative stress. Our results indicated an increase in MDA levels, while the levels of antioxidant enzymes GSH, SOD, CAT, GPx, and GR decreased with age indicating disruption of pro-oxidant-antioxidant balance and loss of physiological function with aging [37, 38]. Administration of BBE in aged rats resulted in a significant decline of lipid peroxidation marked by lowering of MDA levels while provoking an enhanced antioxidant effect witnessed by rising in the levels of GSH, SOD, CAT, GPx, and GR. MDA, being more cytotoxic than reactive oxygen species (ROS), can quickly disrupt cellular activities. GSH on the other hand, reacts with free radicals and peroxides and provides reducing equivalents for GPx and glutathione-S-transferase (GST), which take part in cellular defense mechanisms against intermediate oxygen products [39]. The results observed after blueberry supplementation propound its protective effect against aging *via* neutralization of oxygen free radicals. Several studies indeed suggest a linear correlation between antioxidant activity and the total phenolic concentrations as well as anthocyanins in blueberries [40–43].

Aging is also accompanied by chronic low-grade activation of inflammatory pathways leading to an increase in the production of proinflammatory cytokines [44, 45]. Our results revealed an overproduction of the circulatory inflammatory markers TNF-*α*, IL-6, and NF-*κ*B in liver tissue with advancing age. However, administration with blueberry extract significantly reversed this effect, suggesting its role in suppressing molecular inflammatory pathways. *In vitro* and *in vivo* studies in the past have highlighted the anti-inflammatory and antioxidants effects of blueberry on metabolic impairment. A positive inflammatory response was reported in obese Zucker rats supplemented with BBE powder [46]. The consumption of a diet enriched with wild blueberries significantly declined plasma levels of TNF-*α*, IL-6, and C reactive protein (CRP). Additionally, the expression of CRP declined in the liver while downregulating TNF-*α*, IL-6, and NF-*κ*B in both the liver and abdominal adipose tissues [46]. In human studies, subjects with metabolic syndrome receiving blueberry showed a significant decline in superoxide and total ROS together with a reduction of inflammatory markers and reduced gene expression of TNF-*α*, TLR4, and IL-6 [30, 47]. Although the precise mechanism is not understood, the production of inflammatory cytokines in hepatic tissues as a consequence of aging may be linked to the activation of NF-*κ*B signaling pathway. NF-*κ*B is a key transcription factor of M1 macrophages and is required for the induction of a large number of inflammatory genes, including those encoding TNF-*α* and IL-6 [48]. Thus, blueberry may exhibit its anti-inflammatory effects by suppressing the activation of NF-*κ*B signaling as revealed by our results, although further studies are required to prove this.

Inflammation plays a key role in liver fibrosis development. During fibrosis, macrophages produce profibrotic factors such as the cytokine TGF-*β* and platelet-derived growth factor (PDGF). TGF-*β*, a profibrotic cytokine, plays a crucial role in regulating the different stages of disease development from initial liver injury to fibrosis, cirrhosis, and cancer [49]. Our results indicate the aging increases susceptibility to hepatic inflammation and liver fibrosis as indicated by the rising in TGF-*β* levels in hepatic tissues. It has also been reported that overgeneration of ROS and high glucose levels may contribute to fibrosis development via activation of TGF-*β*1 with resulting production of extracellular matrix proteins. Moreover, our histopathological studies also confirmed a variety of major histopathological changes in liver sections of aged rats, showing enhanced cellular lesions, loss of hepatic tissue structure, and collection of inflammatory cells characteristic of liver injury. Cotreatment with BBE elicited marked declines in cytokine TGF-*β* levels and showed a significant degree of improvement in hepatocyte structure with few atrophied hepatocytes and mild infiltration of inflammatory cells. This data propounds a fibrosis protective effect of blueberry in rat liver. Previous studies have suggested that the preventive mechanism for this effect may be due to inhibition of the expression and activation of NF-*κ*B p65 in hepatocytes, thereby reducing TGF*β*1-mediated production or activation [50, 51].

## 5. Conclusions

In conclusion, our data collectively affirm that dietary administration of blueberry in aged rats significantly improves glycemic control, lipid metabolism, metabolic imbalance, and liver function, while reducing oxidative stress and inflammation in the liver of aged rats. Thus, blueberry acts as a potent antioxidant, anti-inflammatory, and antifibrotic agent against the process of aging. On the basis of these results, blueberries can be used as a feasible dietary supplement for the management and prevention of age-related metabolic disorders and diseases. However, further studies should be conducted in the future to confirm the beneficial effect of blueberry in age-related metabolic disorders and diseases.

## Data Availability

All relevant data are within the paper.
